# Lithium deportment by size of a calcined spodumene ore

**DOI:** 10.1038/s41598-022-22808-7

**Published:** 2022-10-31

**Authors:** Muhammad Kashif Nazir, Laurence Dyer, Bogale Tadesse, Boris Albijanic, Nadia Kashif

**Affiliations:** Western Australia School of Mines, Curtin University, Kalgoorlie, WA 6430 Australia

**Keywords:** Chemical engineering, Engineering

## Abstract

Calcination of spodumene is used to convert α-spodumene to more reactive β-spodumene, has been shown to greatly impact the physical characteristics of some of the components in the ore. This work investigates the energy efficiency of different grinding circuits used for upgrading the lithium content in the finer fraction of the calcined spodumene ore. The results showed that closed-circuit grinding resulted in 89% lithium recovery of the finest size fractions (− 0.6 mm) while open-circuit grinding led to 65% lithium recovery for the same grinding time. Closed-circuit grinding consumed lower energy than open-circuit grinding. The grade of the finest size fraction in the case of the open-circuit grinding was 1.7 times more than that in the case of the closed-circuit grinding. This work shows the potential of using different grinding modes to maximize energy efficiency and lithium deportment by size. However, it is suggested that open circuit grinding should be used for beneficiations of spodumene ores.

## Introduction

Lithium has gained great interest since its discovery in the early 1800s due to its application in glass, ceramic, rubber, grease manufacturing, air conditioning, aluminium industry^1,2^. The recent application of lithium in lithium-ion batteries has resulted in drastic increases in demand^3–6^.

Brine deposits have been the dominant source for lithium extraction in the 1990s being generally more economically favourable. However, lithium content in hard rock lithium ores is higher than that in brine deposits, making the mining of lithium ores a substantial contributor to meet the growing lithium demand^7^.

While there are 145 lithium-bearing minerals, only a few (such as spodumene, petalite, and lepidolite) are commonly used in the production of lithium^8,9^. Spodumene is the most important of all these minerals with a high theoretical Li_2_O content of 8%^2^. Spodumene is a member of the pyroxene group and naturally occurs in α-phase monoclinic form with associated gangue minerals like quartz, albite, microcline and micas in pegmatite deposits^10,11^.

The natural occurring α-phase of spodumene is highly refractory and thus calcination (i.e. thermal treatment at the temperature over 900 °C) is used to convert α-phase to β-phase^12^. The process of calcination makes spodumene more brittle and reactive for acid roasting. The reason is that α-spodumene crystals have a 30% lower volume than β-spodumene crystals. At higher temperatures, the α-phase expands, leading to an increased volume of β-spodumene crystals^13^. Therefore, β-spodumene is less dense than α-spodumene. Previous work^14^ has shown that this expansion induces fracturing between and within spodumene crystals, weakening the particles and facilitating further breakage. This was displayed by a reduction in Bond ball mill work index (BBMWI) of the ore from 44.9 to 25.9 kWh/t after calcination.

Gangue mineral rejections by screening is a very simple method for upgrading a mineral stream based on the propensity of the grade to report to specific size fractions (also known as preferential breakage)^15,16^. When treating raw materials, the behaviour in this regard is driven by the mineralogical association of the valuable element(s), the gangue minerals present and the style of mineralisation (geology). For effective gangue mineral rejections, the gangue minerals must be liberated during breakage. The separability comes from differences in produced particle size of the various minerals during breakage.

Other key component minerals in these samples also undergo phase transitions within the temperature range relevant to calcination. Quartz is well known to undergo a reversible transition from α- to β-quartz at approximately 575°C^17^. The crystal lattice parameters in mica increase at a temperature in the range of 650 °C, which is related to a decrease in the strength of the material^18^. Plagioclase is shown to have reversible changes at approximately 400 °C and irreversible changes at 800–900 °C^19^. Therefore, the properties and response to breakage, handling, and screening will be complex as many components of the ore will be impacted during calcination and grinding. To better understand the physical behaviour of the calcined minerals (ore and gangue) as well as look at other modes of breakage, this work investigates the behaviour of calcined spodumene under both open and closed-circuit grinding.

It should be noted that understanding closed grinding circuits is important for most mineral processing operations for the design and optimizations of grinding circuits. Simulations and scale-up of grinding circuits can be done based on the specific energy to reduce the particle size of the product i.e. the Bond method which could be beneficial for coarse gangue rejection in hard rock lithium ores^20–22^; however, the Bond method may overpredict a mill capacity^23^. The accuracy of grinding circuit simulations can be improved by considering breakage kinetics of valuable minerals, material transport through a mill, and size classification performances^24^. Advances in computational fluid dynamics—discrete element method (CFD-DEM) simulations of grinding circuits may be also used for the design and optimizations of grinding circuits^25^. Although simulations of grinding circuits are very important in mineral processing industries, this is beyond the scope of this work.

## Materials and methods

### Ore preparations

**Figure 1:**
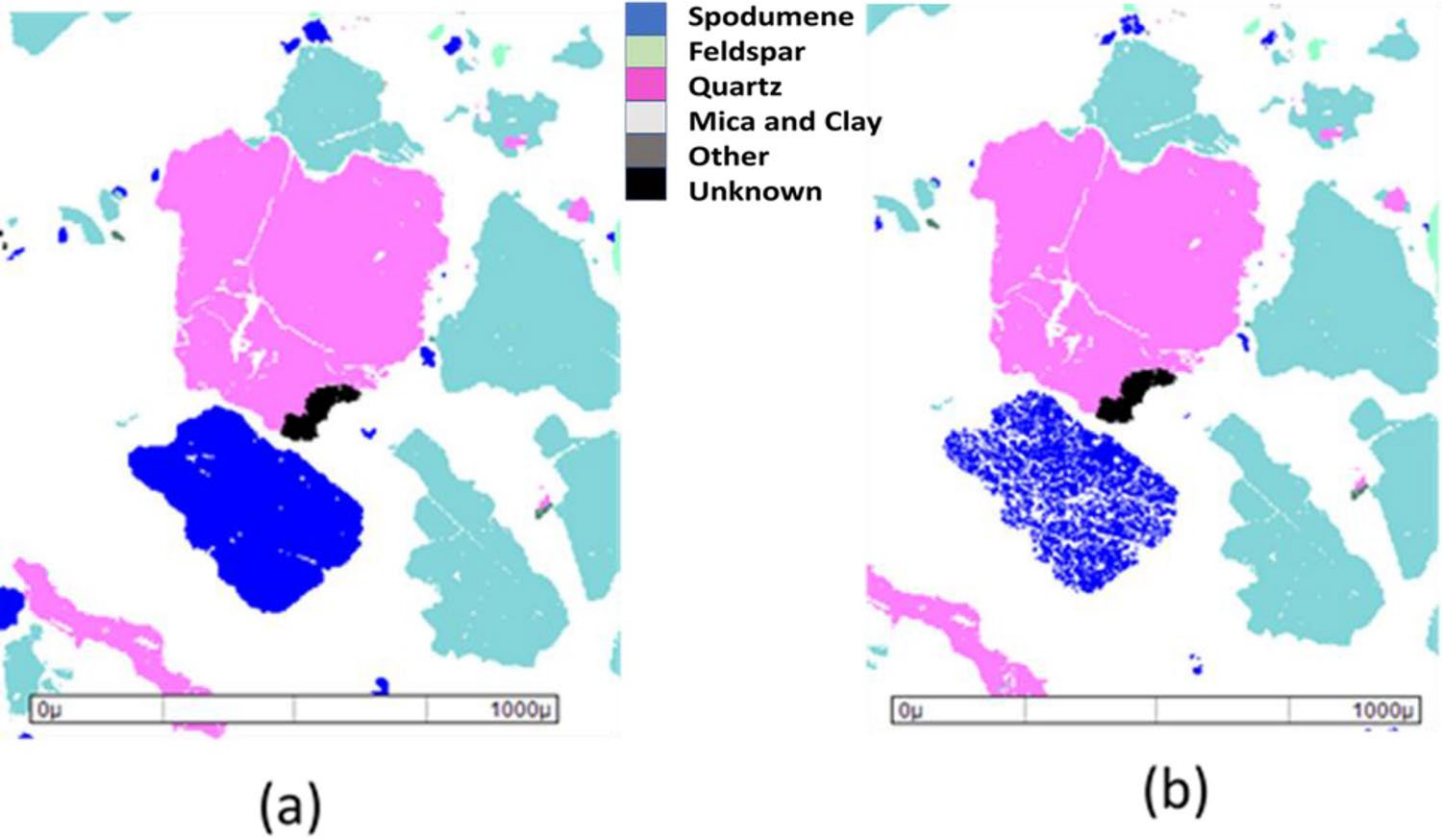
Estimation of particle properties (**a**) before calcination and (**b**) after calcination.

Spodumene ore samples (− 15 mm) were obtained from the Bald Hill Mine Eastern Goldfields, Western Australia. Table 1 shows the mineralogy of the sample determined using XRD. As seen in Table 1, the ore contains spodumene as a valuable mineral with the presence of feldspar, quartz and mica as associated gangue minerals. The ores samples were collected from a low-grade plant feed parcel that has approximately 0.32% Li. The ore (i.e., 3000 g plant feed) was calcined in a muffle furnace (Cupellation furnace, Carbolite Sheffield England) at 1100 °C and held at that temperature for 1 h to ensure complete conversion of α to β-spodumene. After that, the sample was air-cooled slowly to room temperature; the cooling process took 15 h. Other researchers^26,27^ also used the same conditions (i.e., 1100 °C and 1 h calcination time) as used in this study. The muffle furnace temperature was calibrated using the thermocouples with the measurement errors of less than 1.1%. The changes in ore properties after calcination at 1100 °C were characterized by MLA (mineral liberation analyser) and shown in Fig. 1; the MLA software was developed at the University of Queensland in Australia^28^. As seen in Fig. 1, after calcination, spodumene became more porous and hence more brittle.

**Table 1:** Mineralogy of the spodumene ore.

Mineral	% mass
Spodumene	10.6
Feldspar	70.2
Quartz	13.4
Mica	5.5
Others	0.3

The calcined sample was split into two samples to study the influence of different modes of grinding (closed and open-circuit) on coarse gangue rejection and energy consumption of both grinding modes.

### Semi-autogenous grinding and screening

**Figure 2:**
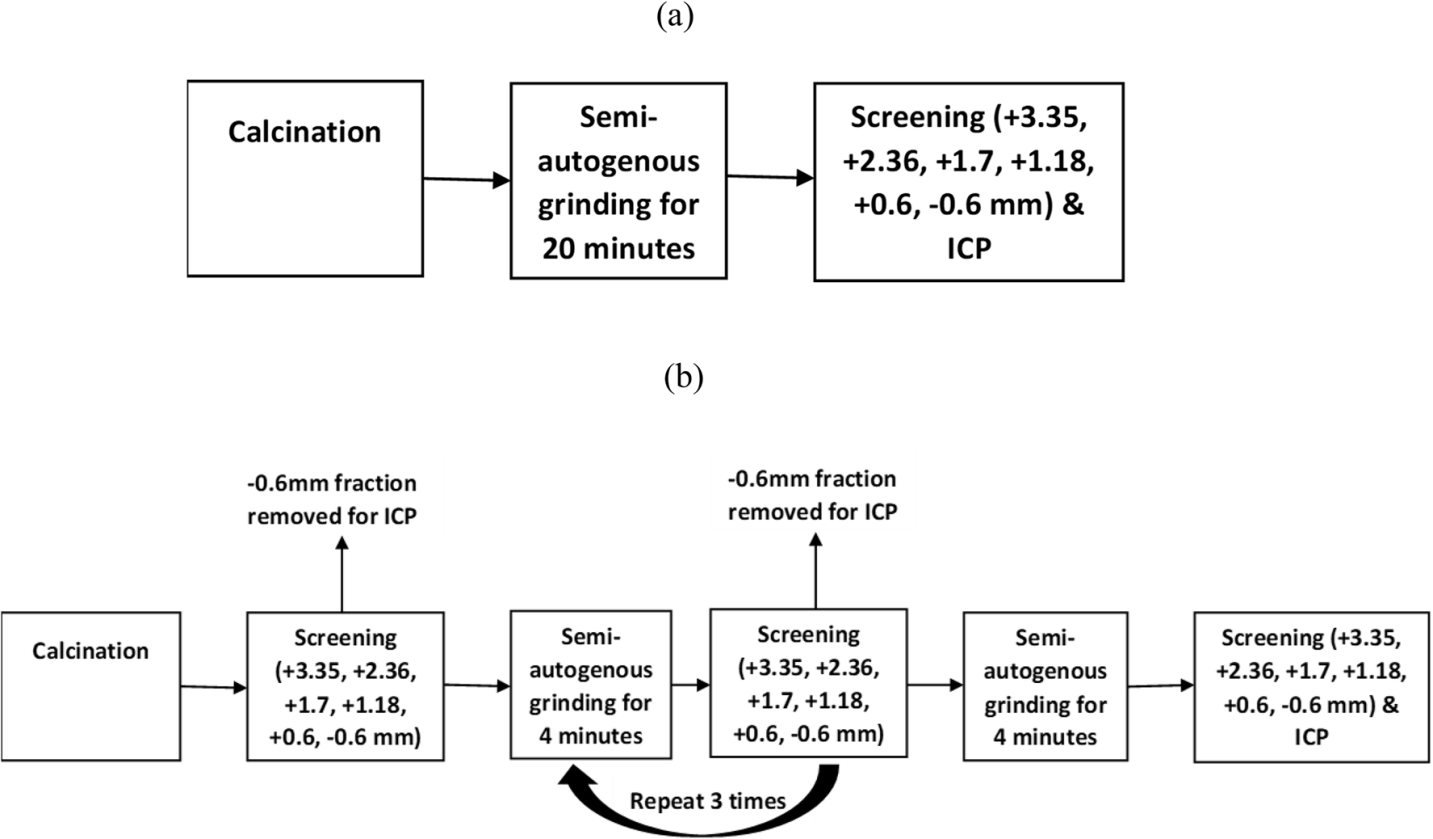
Development flowchart (**a**) the open-circuit grinding and (**b**) the closed-circuit grinding.

Figure 2 shows two flowsheets used in this work. As seen in Fig. 2 both closed and open-circuit grinding was conducted using the calcined spodumene samples. Figure 2a shows that the calcined sample was subject to the open-circuit grinding for 20 min in a ball mill. The ball mill had 12 grinding balls (each grinding ball had 27.3 mm in diameter) with a total mass of 1060 g; the rotational speed of the mill was 70 rpm. The grinding product was screened into six different size fractions (+ 3.35 mm, − 3.35 + 2.36 mm, − 2.36 + 1.7 mm, − 1.7 + 1.18 mm, − 1.18 + 0.6 mm, − 0.6 mm) to study the particle size distribution (PSD) and the lithium grade in the finest fraction (− 0.6 mm). Considering that the ball mill had a low-ball mill loading (10%) in contrast to standard ball milling (50%), the ball mill was used to simulate a semi-autogenous grinding mill.

The calcined sample (Fig. 2b) was also screened into six different size fractions (+ 3.35 mm, − 3.35 + 2.36 mm, − 2.36 + 1.7 mm, − 1.7 + 1.18 mm, − 1.18 + 0.6 mm, − 0.6 mm) to determine the particle size distributions of the feed. This sample was ground using the closed-circuit grinding in the same ball mill used for the open-circuit grinding to compare this mode of grinding with the open-circuit grinding.

As seen in Fig. 2b, there are 5 grinding stages, and each grinding stage was 4 min i.e. the overall time for grinding was 20 min. After each grinding stage, the calcined sample was screened into two different size fractions: − 0.6 mm (the fine fraction) and + 0.6 mm (the coarse fraction). The fine fraction was not ground again to prevent overgrinding of the feed and thus reduce energy during grinding. The coarse fraction was ground after each grinding stage. The coarse fraction was screened into five different size fractions (+ 3.35 mm, − 3.35 + 2.36 mm, − 2.36 + 1.7 mm, − 1.7 + 1.18 mm, − 1.18 + 0.6 mm) to study the PSD (particle size distribution) after each grinding stage. The chemical analysis was conducted through digestion of the solids, and ICP-OES (Agilent Technologies 5100) was used to determine the lithium content in the fine size fraction (− 0.6 mm). The standard deviation for three repeats of the lithium grade did not exceed 3%.

### X-ray diffraction (XRD)

Mineralogical analyses of the lithium ore samples were conducted using an Olympus BTX™ III Benchtop (Co-Kα) XRD with radiation Co-Kα in the range between 5 and 55° (2θ). XPowder from Olympus was used for analyzing the diffractogram. The XRD experiments were performed using six calcined finest size fractions collected after each grinding stage to identify the changes in the mineralogy of spodumene after grinding. These experiments were also conducted for the finest size fraction collected after the open-circuit grinding. The XRD trends were detected via visible inspection of the peak intensity and shape and there is a notable change in relative intensities which correlates well with the assays.

## Results and discussions

### Effect of grinding time on particle size distributions of feed in open and closed-circuit grinding

**Figure 3:**
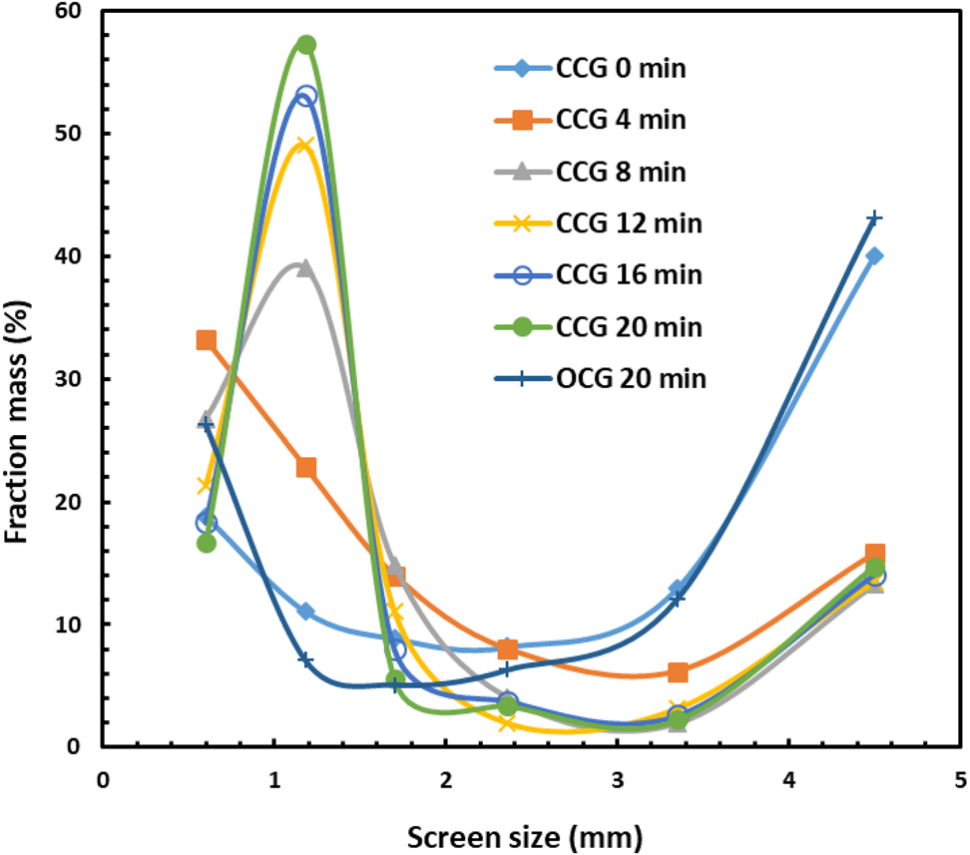
Particle size distributions as a function of grinding time (CCG is the closed-circuit grinding and OCG is the open circuit grinding).

Figure 3 shows the influence of grinding time on particle size distributions. As would be expected the overall PSD (i.e. particle size distribution) after each additional stage of grinding is shifting to finer fractions. However, it must be noted that after each grinding stage the − 0.6 mm fraction is removed from the system, therefore in each subsequent stage, a greater proportion of fines is generated with the same energy input. Again, this is expected as with each subsequent stage there is less sample mass in the mill.

Figure 3 also compares the particle size distribution for the open-circuit grinding and that for the closed-circuit grinding. As seen in Fig. 3, the open-circuit grinding produced significantly more coarse particles (− 3.35 + 2.36 mm) than the closed-circuit grinding. The reason is that during the closed-circuit grinding, the fine particles (− 0.6 mm) were removed from the mill every 4 min of grinding while during the open-circuit grinding the ore remained in the mill for 20 min. Therefore, the closed-circuit grinding after 20 min produced 8.3 times more fine particles (− 0.6 mm) than the open-circuit grinding. The removal of fine particles during closed-circuit grinding makes it possible for new materials to have direct contact with the grinding media which enhances the grinding, hence generating more fine particles. Removing fine and softer material allows breakage for harder minerals. Nevertheless, the open-circuit grinding produced 27% of ore particles less than 0.6 mm while the closed-circuit grinding after the fifth stage produced 17% of the same size fraction.

### Effect of grinding time on lithium deportment in closed-circuit grinding

**Figure 4:**
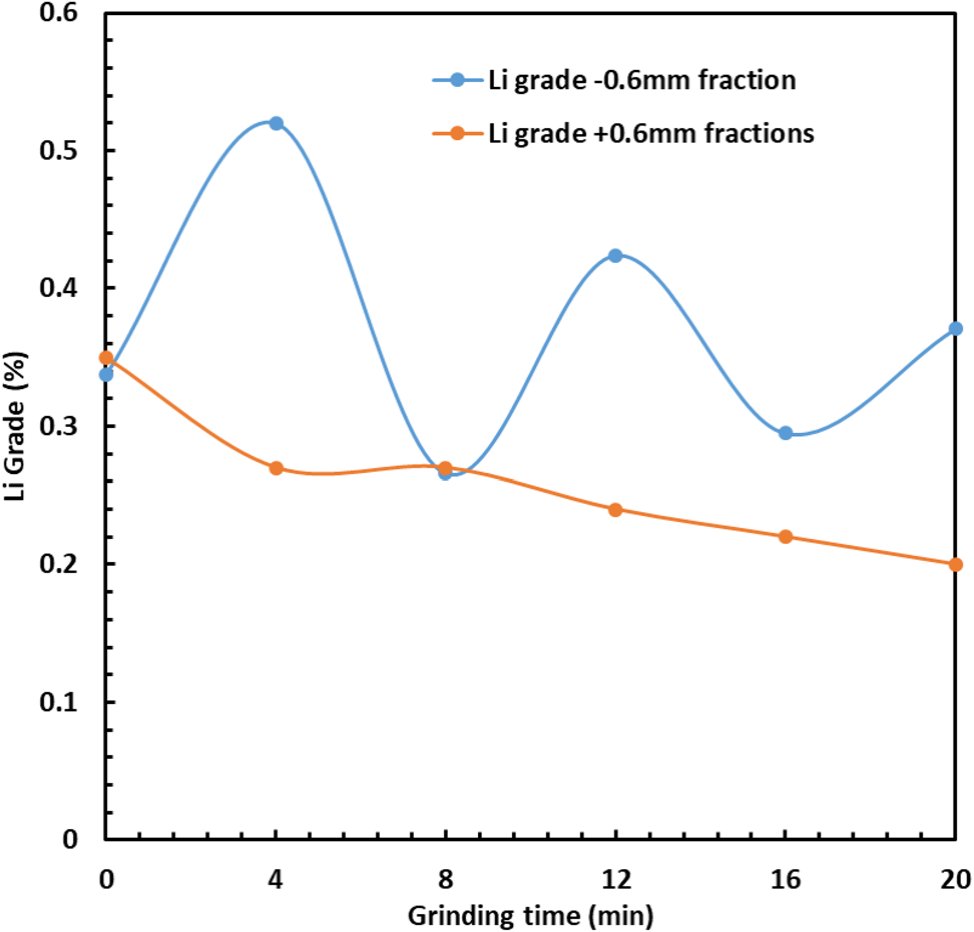
Effect of closed-circuit grinding time and screening on lithium grade in the two size fractions: − 0.6 mm and + 0.6 mm.

Figure 4 shows the changes of Li grade for the − 0.6 mm fraction during closed-circuit grinding. As seen in Fig. 4, the Li grade changed significantly after each 4 min grinding interval resulting in two maxima and two minima. The first maximum occurred after 4 min due to preferential grinding of the brittle β-spodumene. In the second stage of grinding (i.e. the first minimum), after removal of the − 0.6 mm fraction, the lithium grade decreased because the β-spodumene was not completely deported in the finest size fractions, and the removal of fines induced breakage of coarser minerals; it means that spodumene was occluded in the gangue. In the third stage (i.e., the second maximum), Li grade in the finest size fraction increased due to β-spodumene liberation from gangue minerals. In the fourth stage, the same trend was also observed as in stage two. Finally, in the last stage, the Li grade increased, which is similar to stage three; however, the Li grade in the last stage was 69% less than the highest Li grade achieved (i.e. the first stage of grinding); the Li grade after the fifth stage was 0.38%. It should be noted that the Li grade (− 0.6 mm) after the open-circuit grinding was 0.65%. These results showed that the closed-circuit grinding resulted in oscillating of Li grade with different grinding times. The progressive depletion of Li in the residual coarse fraction (+ 0.6 mm) is also shown in Fig. 4.

It should be noted that Fosu et al.^26^ found that calcined spodumene is highly liberated when P_80_ was between 50 and 80 µm. In our work, P_80_ was between 600 and 800 µm (see Fig. 8), indicating that spodumene was less liberated. However, Fig. 1 shows that the sample of spodumene is fully liberated, and Fig. 1 did not show unliberated spodumene. However, based on the results obtained in Fig. 4, spodumene was occluded. Further research is required to investigate the liberation degree of calcined spodumene as a function of grinding time.

### XRD analysis of the finest size fractions (− 0.6 mm) of open and closed circuit grinding

**Figure 5:**
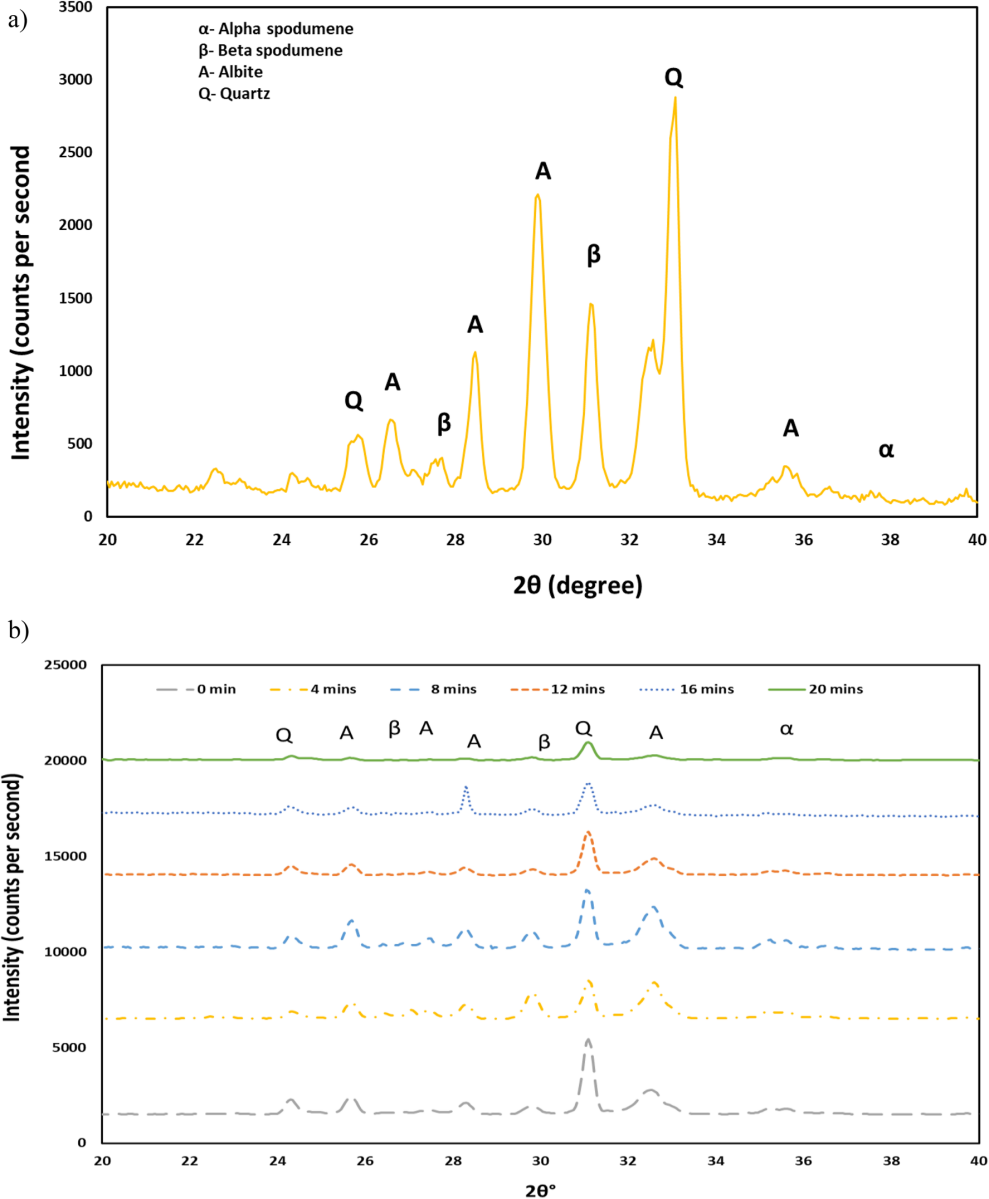
XRD results for samples collected after (**a**) the open-circuit grinding and (**b**) the closed-circuit grinding.

The mineralogy of the grinding products for the finest size fractions (− 0.6 mm) obtained after the closed-circuit and open-circuit grinding is shown in Fig. 5. The XRD analysis for the open circuit grinding (see Fig. 5a) shows that a significant amount of β-spodumene was deported to the finest size fraction, leading to the lithium grade of 0.65% which is higher than that achieved after the fifth stage of the closed-circuit grinding (i.e. 0.38%).

Figure 5b shows the XRD analysis conducted to investigate the changes in the mineralogy of the products after each stage of the closed-circuit grinding. The amount of quartz was the highest before grinding. However, the amount of β-spodumene was the highest after the first stage of grinding which agrees well with the maximum lithium grade shown in Fig. 4. The amount of albite and quartz was large after the second and fourth stages of grinding, leading to a lower lithium grade (see Fig. 4). The reason is that albite has irreversible changes at 800–900 °C^19^, resulting in a higher crystal lattice angle. The amount of albite after the third and fifth stages of grinding was lower than that after the second and fourth stages of grinding, resulting in a higher lithium grade after the third and fifth stages of grinding. These results showed that the XRD analysis is a useful method for understanding the changes in ore mineralogy after the closed-circuit grinding of calcined spodumene samples.

### Lithium recovery in closed-circuit grinding

**Figure 6:**
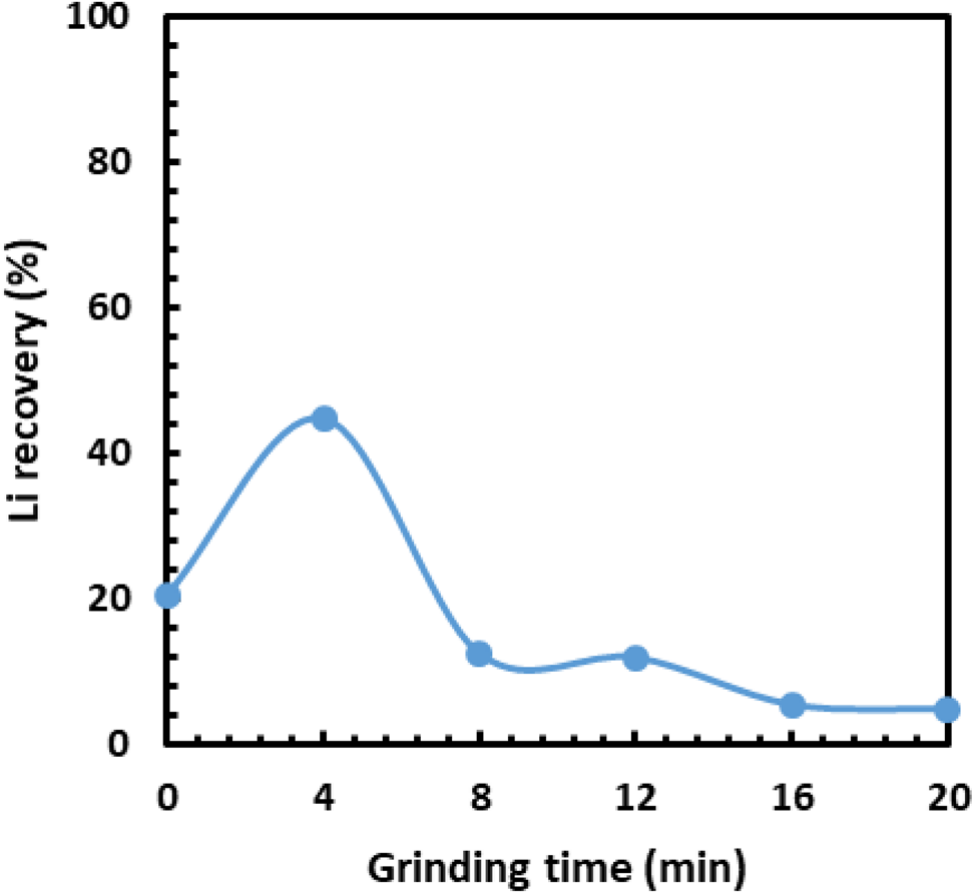
Effect of grinding time and screening on the lithium recovery in the finest size fractions (− 0.6 mm).

Figure 6 shows the changes in Li recovery for the finest size fraction during closed-circuit grinding. As seen in Fig. 6, the maximum Li recovery was achieved after the first stage of grinding (i.e. after 4 min) because the maximum amount of β-spodumene (i.e. very brittle mineral) was deported to the finest size fraction. However, in the second stage of grinding, the Li recovery dropped considerably, indicating that the amount of β-spodumene was significantly reduced. This trend was also observed after the third, fourth and fifth stages of grinding due to the decreasing remaining mass of the ore in the mill.

**Figure 7:**
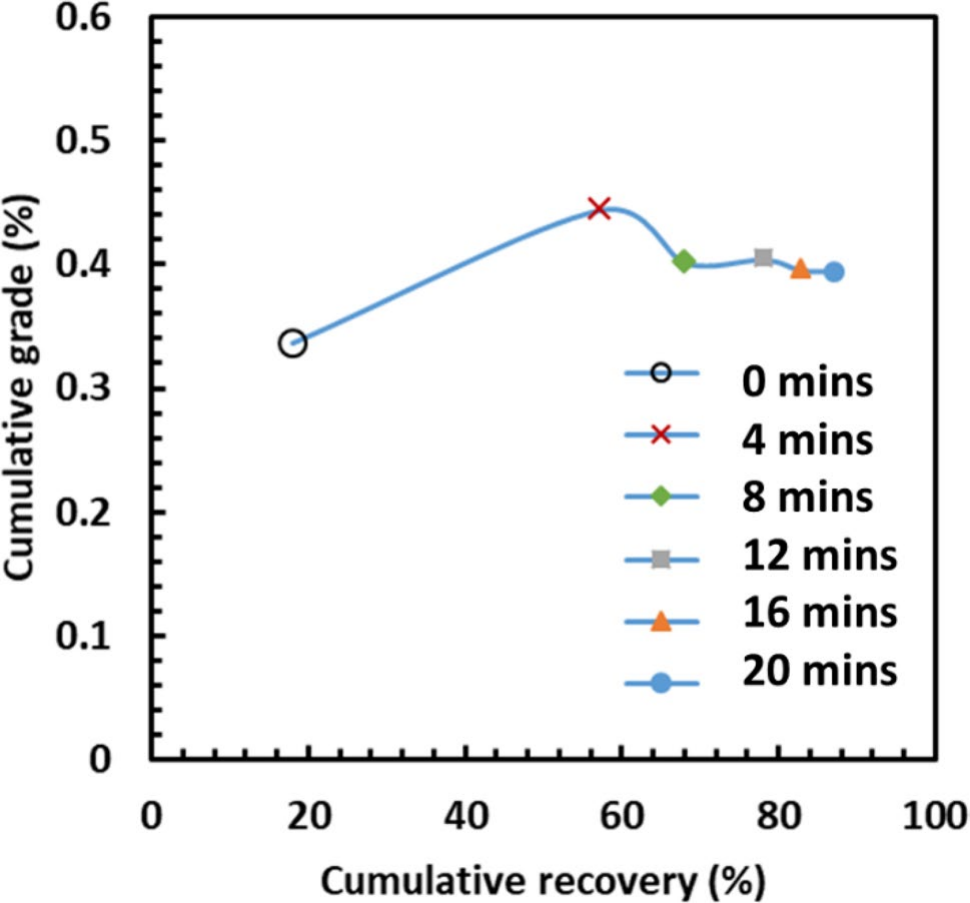
Cumulative recovery vs cumulative grade for the finest size fractions during the closed-circuit grinding.

Figure 7 shows the relationships between the cumulative Li grade and the cumulative Li recovery at different grinding stages. As seen in Fig. 7, the maximum Li grade and Li recovery were obtained after the first stage of grinding (i.e., after 4 min) because β-spodumene was deported in the finest size fraction. In the subsequent grinding stages (the second, third, fourth, and fifth stage), the oscillating relationship between the cumulative Li grade and the cumulative Li recovery was observed, indicating that the deportment of Li in the finest size fraction changes with every grinding stage.

### Comparison of the efficiency of the closed and open-circuit grinding

**Figure 8:**
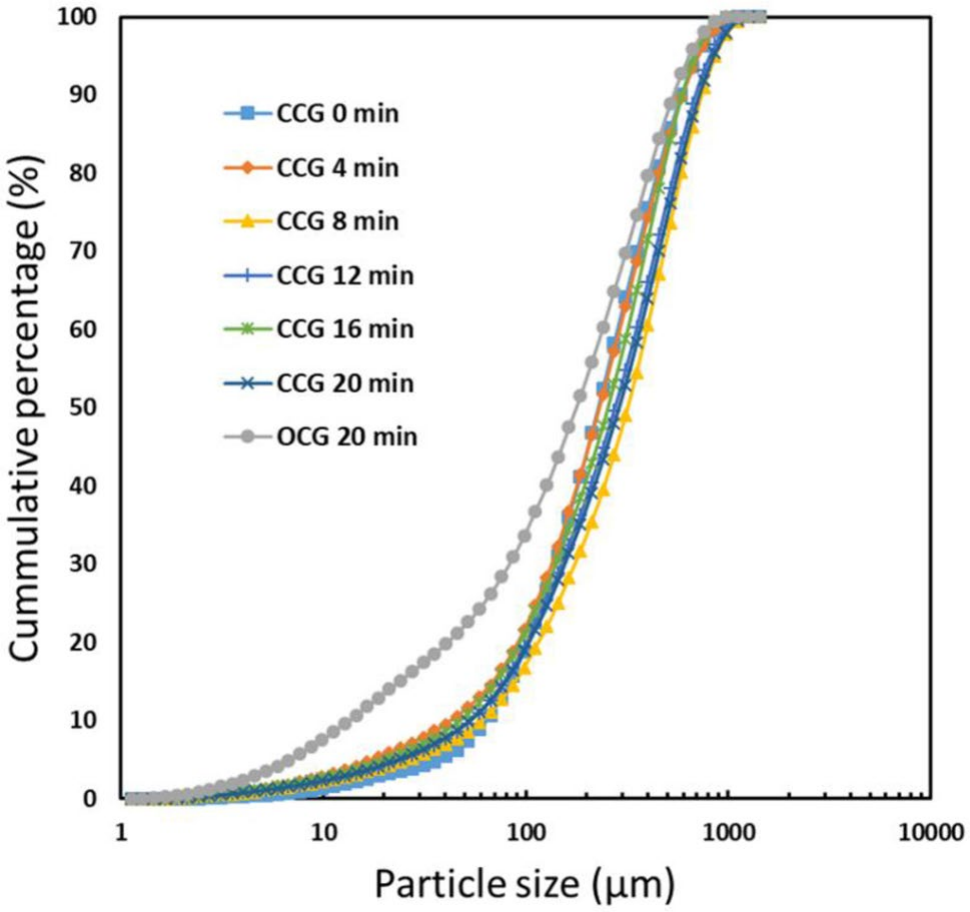
The particle size distributions of the products (− 0.6 mm); CCG is the closed-circuit grinding and OCG is the open circuit grinding.

Table 2 compares the efficiency of the closed and open-circuit grinding. The results showed that in the case of the finest size fraction (− 0.6 mm), the mass recovery for the closed-circuit grinding was 77% while that for the open-circuit grinding was 26% (i.e. the closed-circuit grinding generated 1159 g of the finest fraction whereas the open-circuit grinding generated 389 g; the total mass was 1482 g). The reason is that after each increment of closed-circuit grinding, the finest size fraction was removed from the system, leading to enforced grinding of the coarse material. In the open-circuit grinding, the softest components are preferentially ground and then overground generating an overall finer PSD (Fig. 8) and leaving the harder components in the coarse fraction. The closed-circuit grinding also led to a 24% higher lithium recovery than that of the open-circuit grinding. It means that the closed-circuit grinding generated 3 times finer fractions than open-circuit grinding. This work showed that the closed-circuit grinding is more energy efficient than the open-circuit grinding due to the generation of more finer fractions during the closed-circuit grinding, using 3 times less energy per unit mass of fines produced.

**Table 2:** Mass balance and energy consumption for both open and closed grinding circuit.

Cycle number	Cycle duration (min)	Mass (feed) g	Mass (product − 0.6 mm) g	Energy consumption per ton of product (kWh/t)
**Closed-circuit grinding**				
1	0	1482	279	
2	4	1199	398	130
3	8	799	214	70
4	12	585	125	41
5	16	458	84	27
6	20	355	59	19
**Open-circuit grinding**				
1	20	1492	389	857

However, in the finest size fraction, the Li grade of the products in the case of the closed-circuit grinding was 56% less than that of the open-circuit grinding. The reason is that the particle size in the − 0.6 mm size fraction produced by the open-circuit grinding was lower than that produced by the closed-circuit grinding as seen in Fig. 8. Thus, finer particles were deported in the finest size fraction (− 0.6 mm) during the open-circuit grinding than during the closed-circuit grinding, leading to a higher lithium grade during the open-circuit grinding. Similar observations were obtained by Peltosaari et al.^27^ using autogenous grinding of the calcined spodumene.

Table 2 shows the mass and energy balance for both open and closed grinding circuit. Table 3 compares the total performance of both open and closed grinding circuit. As seen in Table 3, the closed-circuit grinding led to 89% lithium recovery while the open-circuit grinding resulted in 65% lithium recovery. The energy consumption during the open-circuit grinding was 3 times higher than that during the closed-circuit grinding. It should be noted that the energy consumption was determined using the motor power of 1 kW, the grinding time of 0.33 h, and the mass of the sample produced in the finest size fraction (− 0.6 mm) (i.e. the overall mass of the finest size fractions in the closed-circuit grinding was 1159 g and that in the open-circuit grinding was 389 g). The energy consumed (E) during grinding is determined using Eq. (1):

1 $$ E^{*} = \frac{E}{m}t $$

E^*^ is the consumed energy in kWh/t, E is the energy in kW, m is the mass of the product in the fine size fractions (− 0.6 mm); t is the time (h).

**Table 3:** Comparison of the closed-circuit grinding versus the open-circuit grinding.

Grinding type	Product particle size (μm)	Li grade (%)	Mass recovery (%)	Li recovery (%)	Energy consumption (kWh/t)
Open-circuit	− 0.6	0.65	26%	65%	857
Closed-circuit	− 0.6	0.38	77%	89%	287

This work demonstrated that the closed-circuit grinding should be used for maximizing Li recovery and minimizing energy consumption while the open-circuit grinding should be used for maximizing Li grade. These results demonstrated the benefits of the closed-circuit grinding for a significant energy reduction per ton of product due to the elimination of overground fines, which agrees well with the literature^29,30^. This grinding mode also increased the Li recovery.

## Conclusions

This work investigates the behaviour of calcined spodumene ore using different modes of grinding. The results showed that the maximum amount of β-spodumene (i.e., very brittle mineral) was deported to the finest size fraction (− 0.6 mm) after the first stage of grinding (i.e., after 4 min), resulting in the maximum Li grade and recovery at this grinding stage. Although the lithium recovery was almost constant in the next four grinding stages, the lithium grade oscillated after each grinding stage. The results showed that there was greater preferential deportment of Li to the fines in open-circuit grinding, the recovery was lower due to the much lower fine generation. As would be expected, closed-circuit grinding is far more efficient in generating fine material but is far less discriminatory between phases as the softer material is ground faster and removed leading to the harder components of the ore being ground over time. Whereas, open-circuit grinding continues to preferentially grind the softer material generating much finer particles at the lower end of the size distribution. The XRD analysis of the samples shows that the hardness of other materials such as albite may also be impacted by calcination. This work shows the potential for selection of milling process and conditions to optimise Li deportment to the fines in calcined material.

## Data Availability

All data generated or analysed during this study are included in this published article.
